# History of creatinine clearance: tribute to a forerunner

**DOI:** 10.1093/ckj/sfad024

**Published:** 2023-02-06

**Authors:** Attilio Losito

**Affiliations:** Renal Unit, Ospedale Santa Maria Della Misericordia, Piazzale Menghini, Perugia, Italy

**Keywords:** creatinine, creatinine clearance, history of nephrology, kidney function, pioneers in medicine

## Abstract

It is known to few that the path to the discovery of the long-denied plasma creatinine and its clearance was long and difficult. For quite a long time, related controversies between different groups of researchers were widespread and heated. The scientists who have dealt with the related problems are among the most famous of the last century and some of them are part of the history of medicine. Giovanni Ferro-Luzzi, an Italian clinician, was one of these researchers. He was among the first to detect and dose plasma creatinine and the first ever to measure the clearance of endogenous creatinine (CrCl). Unfortunately, due to a series of unforeseeable events, he has been completely forgotten together with his undertakings. In this review we retrace the steps that led to the measurement of plasma creatinine, and CrCl. With brief biographical notes we try to explain the oblivion of this important figure and of his nephrological accomplishments.

## BACKGROUND

Endogenous creatinine clearance (CrCl) is nowadays a widely accepted and validated renal function test for clinical purposes. However, not everyone knows that the path to reaching its formulation and acceptance has been full of obstacles and controversies. The history of its discovery is largely forgotten as are some of the people who contributed to it. These were outstanding figures whose achievements were not limited to the complex history of creatinine and its renal clearance but also extended to other fields of medicine. One of these scientists is the subject of our account. His scientific accomplishments, brought to light recently, are intertwined with some of the tragic events of the past century [1].

## THE CREATININE CONTROVERSY

Arthur R. Cushny's (1866–1926) theory, which he termed the ‘modern view’, stated that ‘secretion (a pure filtration) occurs at the glomerulus’ [2]. Once the concept of filtration had been established, the method by which to measure it remained to be identified. Studies using urea for the evaluation of glomerular filtration were based on complex calculation formulas and results were invalidated by its tubular excretion [3–5]. In 1926 the Danish animal physiologist Poul Brandt Rehberg (1895–1989) tried a different marker: creatinine. In his experiment, he administered an oral load of 5 g of creatinine to attain measurable plasma concentrations, around 8 mg/dL, and collected hourly urines to measure volumes and creatinine content. He applied the formula $F = \frac{Au}{Ap}\ast U$, were *Au* represents urinary creatinine, *Ap* its plasma concentration and *U* urine volume [6].

Unfortunately, the administration of creatinine induced high plasma values with consequent tubular excretion that produced falsely high clearance values. Nevertheless, Rehberg's formula represents the first formal calculation of glomerular filtration rate with creatinine and coincides with what we call now a ‘clearance’ measurement, a term then unknown. Later, Rehberg's experiment was reproduced by others who obtained comparable results [7]. Why in Rehberg’s study did creatinine have to be infused? To answer this question we must go back to one of the most debated scientific issues of the past century concerning the actual presence or the measurability of creatinine in plasma. Only one year after the chemical synthesis of creatinine, the German pharmacologist, Max Jaffé (1841–1911), discovered a reaction of creatinine with picric acid in an alkaline environment [8]. After a few years, Otto Folin (1867–1934), an already famous professor of biological chemistry at Harvard University, devised a new method to detect and measure small amounts of creatinine in biological fluids [9]. Soon after this discovery several researchers were confronted with this measurement but the results were mixed. Stanley Benedict (1884–1936) published an extensive series of observations on the Jaffé-reactive material in blood. These relatively non-specific techniques elicited some striking differences between pure creatinine and the chromogenic substance in blood filtrates. He therefore concluded that ‘creatinine does not exist in blood in detectable quantities’ [10]. Other leading scientists supported these conclusions [11]. The heated controversy then moved from the USA to Europe [12], where, along with biochemists, clinicians also tackled the issue. Among these emerges the figure who is the subject of our account, the Italian Giovanni Ferro-Luzzi (FL) (1903–2000). With a series of experimental and clinical researches he directly challenged Benedict's denial theses. In fact, FL devised a modification of Somogyi's original method for the preparation of blood filtrates [13]. Thanks to this procedure he was able to eliminate chromogens from the serum by a complex precipitation technique that spared the creatinine. FL could thereby prove that creatinine is present in human blood and can be accurately measured. He published the details of all his experiments in a prestigious German scientific journal [14]. It is remarkable that the creatinine values obtained by his method (0.6–1.4 mg/dL) are in good keeping with those verified later with more modern techniques and are in line with today's standards. Moreover he considered plasma creatinine not only a measure of renal function but also a prognostic index [15]. The creatinine controversy was finally resolved years later by the introduction of a very specific enzymatic method by Dubos and Miller [16]. They concluded that ‘this method gives creatinine values which are equivalent to those obtained by the alkaline picrate method’, making a reference to the FL paper of 1935.

## FIRST MEASUREMENTS OF THE CLEARANCE OF ENDOGENOUS CREATININE

Thanks to this ‘discovery’, FL was able to use the plasma creatinine, obtained with his technique, to measure the glomerular filtration rate. He implemented Rehberg's formula but without administering the oral creatinine load. He entered instead into the formula the plasma creatinine values obtained by his method. The results were outstanding and were soon published [17]. The values of glomerular filtration rate obtained in healthy subjects in steady state conditions were between 88.3 and 165.3 mL/min (mean value 112.6), quite consistent with current standards. The title of the paper was ‘Kidney function in the light of modern views; studies on tubular reabsorption’. To our knowledge this is the first report of a measurement of GFR by the CrCl (Table 1). In 1935, FL reported new experiments conducted on CrCl measured with his improved method [18]. Surprisingly, the implementation of CrCl, the main subject of the studies, did not appear in the title of either article. This factor may later have contributed to obscuring the innovation brought by these studies. At the time, acknowledgement of FL’s accomplishment came soon from the Austrian physician Hans Popper (1903–88) in Wien. This famous figure, before moving to the USA in 1938, due to the political climate, and becoming the father of modern hepatology, at the beginning of his career in Wien had devoted himself to kidney studies [19]. In 1937, Popper published the landmark paper of 110 pages, which can more appropriately be called a monograph, on CrCl [20]. The paper was appropriately entitled ‘Filtration and reabsorption in renal pathology’. From this outstanding and extensive study he drew the general credit for introducing this clinical test of renal function [21]. Yet, in his paper Popper cites the 1934 and 1935 articles of FL, stating ‘Only a few authors have carried out the investigations without creatinine load; FL gives filtrate values between 88.3 and 165.3 mL/min, on average 113.4. FL, Saladino and Santamaura, which use an improved creatinine determination method, have a larger material and find similar values to ours’. In his references Popper does not limit himself to citing the three works in German by FL but also cites the paper given at the congress of the Italian Society of Internal Medicine on ‘Renal function in diabetes’, thus demonstrating that he thoroughly followed the development of FL's research [22]. With this quote Popper actually recognizes not only the absolute priority of the Italian author in the ClCr implementation, but also the innovative method he devised for the measurement of plasma creatinine. The acknowledgement was reiterated a few years later. Popper, now in the USA, in an article on the relation between creatinine and urea clearance in renal disease, stated again that ‘Ferro-Luzzi published data concerning the endogenous CrCl’, quoting the 1934 paper [23]. Until 1940, FL’s studies, addressing almost exclusively the kidney, were widely quoted not only in medical journals but also in textbooks [24–26]. Then, almost suddenly, this enterprising scientist, pioneer in the studies of kidney function, disappeared from the nephrological scene, where his name, with very few exceptions, was afterwards completely forgotten [27]. To find an explanation to this forgetfulness we must address FL’s biography.

**Table 1:** Main stages in the discovery of endogenous creatinine clearance.

Author	Year	Contribution	Ref no.
Detection and measurement of plasma creatinine			
Jaffé	1886	Reaction of creatinine with picric acid	[8]
Folin	1914	Application of a colorimetric method to Jaffé reaction	[9]
Ferro-Luzzi	1934	Detection of plasma creatinine with Somogyi's method	[14]
Zacherl and Lieb	1934	Photometric measurement of plasma creatinine	[12]
Dubos and Miller	1937	Enzymatic measurement of plasma creatinine	[16]
Creatinine clearance implementation			
Rehberg	1926	Clearance of esogenous creatinine (oral load)	[7]
Ferro-Luzzi	1934	Clearance of endogenous creatinine	[17]
Popper	1937	Reliabilility and reproducibility of endogenous creatinine clearance in health and in disease	[20]
Steinitz	1940	Suitability of endogenous creatinine clearance for the determination of the glomerular filtration (comparison with inulin clearance)	[24]

## BIOGRAPHY OF GIOVANNI FERRO LUZZI

FL was born in Ancona in 1903, and was educated in Rome where he graduated in Medicine in 1928 and where he started his medical career. In 1930 he got married to Sofia Salzmann (1903–63) a Russian doctor of Jewish descent with whom he had four children, destined to become top researchers in various branches of science. From 1932 to 1935, FL split his time between Rome and Messina, where he had developed a collaboration with Carmelo Ciaccio (1877–1956), a pathologist of the local university renowned for his biochemical research. There he developed his method for measuring plasma creatinine.

**Figure 1:**
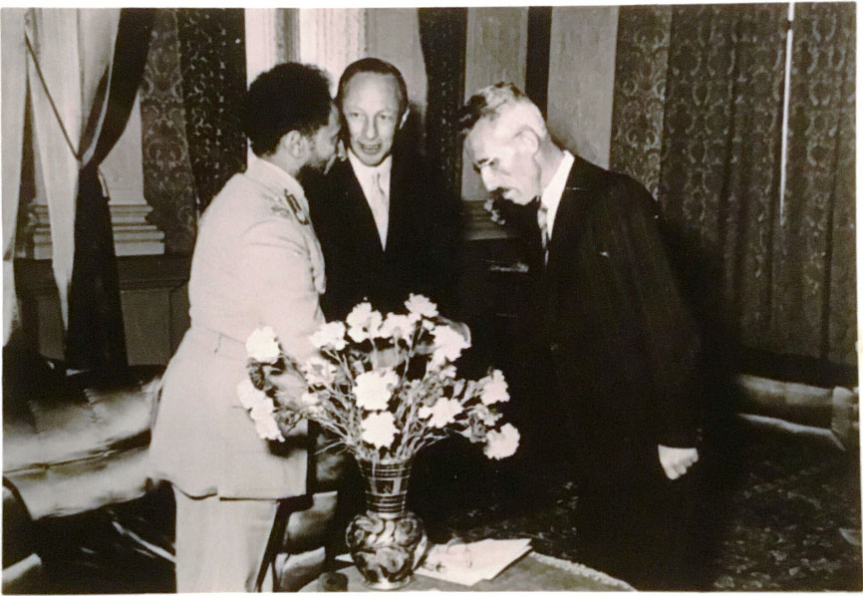
On the left is shown the title page of Ferro-Luzzi's 1934 paper, the first ever description of endogenous creatinine clearance. The right inset is the table from the same paper showing under the heading ‘F_1_ ccm’ the values of glomerular filtration in healthy subjects.

In 1935, FL became deputy director of the medical unit of the San Camillo Hospital in Rome where he continued his kidneys studies. The turning point in his life occurred in 1938. In that year, the Italian fascist government enacted the racial laws that banished Jews from their professional positions. These laws directly affected FL's family through his wife who, as a researcher at the Italian National Research Council, was consequently expelled. These facts and the general political climate prompted FL to take the drastic decision to leave Italy, in a singular coincidence with Popper's flight from Austria. Thus, he applied for and was given the position of director of the Internal Medicine Unit of the ‘Ospedale Regina Elena’ of Asmara, Eritrea. In 1939, he was also appointed director of the whole hospital and, in 1941, during the British occupation of Eritrea, FL was charged with establishing and directing the ‘Medical School of Asmara’, which he himself had conceived. The prestige enjoyed by the school is testified not only by many of its students but also by the trust placed in his directorship by large swathes of the population. Among these stands out Hailé Selassié, at the time Emperor of Ethiopia, who sought FL for a medical opinion (Fig. 1) [28]. A remarkable feat of that period was his participation in a scientific expedition in Dankalia, whose main purpose was to observe the feeding conditions of the nomadic populations who lived in those arid lowlands with very high temperatures (up to 48°C). The results of the expedition yielded an important and full-bodied monograph that still serves as a reference today [29].

The move to Africa and the consequent organizational commitments had not quenched FL's spirit of research; instead he adapted his interests to the new reality, where Kala-azar, Bilharziasis and other local conditions were the prevalent issues. Most of all, in his view, the population's nutritional problems were the most challenging. Strengthened by this conviction, he addressed the issue. From that time, nutrition in developing countries became his main field of scientific interest, making FL again a forerunner in medicine. An anecdote related to this field of research provides insight into FL's investigative spirit. In 1936, serving as a doctor assigned to a military expedition in Ethiopia, he noticed that indigenous troops during their long stay in the desert, despite a diet devoid of fresh vegetables and fruit, showed no signs of vitamin deficiency. FL tried to examine the foods of local soldiers. Despite the lack of an equipped laboratory, aware of the recently discovered method for detecting ascorbic acid, he used the methylene blue of his fountain pen and the strong desert irradiation for a makeshift test [30], and was thereby able to detect the presence of vitamin C in Berberè powder, a spice used in soldiers’ food (episode reported personally by his daughter, professor Anna Ferro-Luzzi). During the African years, FL carried an extensive work. This represents his legacy for that territory, and his role in evaluating and improving the nutrition of African peoples is still widely recognized. As recently as in 1988, the official publication of the Ethiopian ministry of health still cites 48 papers by FL on food and health of the local population [31, 32]. Even when in 1955 his African experience ended, due to growing political instability in Asmara, FL's research activity continued. He returned to Rome, as director of the Nutrition Service of the Ministry of Health, where his efforts were now directed towards the great nutritional issues at home and abroad. He developed an international cooperation, under the aegis of the World Health Organization and the Food and Agriculture Organization, with surveys of the state of nutrition in various developing countries. His original investigative methods, in Italy, Libya, Somalia, Morocco, Mauritania and French Polynesia, yielded remarkable results [33–37]. After retirement in 1968, FL turned his interests to painting and travel. He deepened his knowledge of Indian culture, customs and religion with long and adventurous travels. He remained active and full of interest until the end of his days in 2000 at 97 years old.

The forced distance for many years from the European scientific scene is certainly the main cause of the progressive oblivion of FL's nephrological feats together with the loss of value and diffusion of German periodicals following the Second World War. Yet, we understand that nephrology, his first scientific interest, forcibly abandoned, was still with him also after many years. In fact in 1954, he wrote again a nephrological paper [38]. In the article he claims his original method for calculating CrCl, without creatinine load, and states that for about 20 years he had used it successfully in kidney diagnostics. We can only assume that this was the best way a man of great dignity could express his regret for the general forgetfulness of his nephrological breakthroughs. We hope that with this report on the history of CrCl and the role played by FL we can contribute to restore to this scientist his deserved place in the history of nephrology.
